# A Case Report of Deep Gluteal Syndrome Secondary to Piriformis Enthesopathy Successfully Treated With Ultrasound-Guided Dextrose Prolotherapy

**DOI:** 10.7759/cureus.102370

**Published:** 2026-01-27

**Authors:** Yonghyun Yoon, Dongyeun Sung, Jonghyeok Lee, King Hei Stanley Lam

**Affiliations:** 1 Orthopedic Surgery, Kangnam Sacred Heart Hospital, Hallym University, Seoul, KOR; 2 Orthopedics, Incheon Terminal Orthopedic Surgery Clinic, Incheon, KOR; 3 Neurosurgery, Himchannamu Neurosurgery Clinic, Daegu, KOR; 4 Neurosurgery, Bareun Neurosurgery Clinic, Cheongju-si, KOR; 5 Faculty of Medicine, The Chinese University of Hong Kong, Shatin, The New Territories, HKG; 6 Faculty of Medicine, The University of Hong Kong, Hong kong, HKG; 7 The Board of Clinical Research, The Hong Kong Institute of Musculoskeletal Medicine, Kowloon, HKG

**Keywords:** deep gluteal syndrome, dextrose prolotherapy, dynamic examination, enthesopathy, piriformis, sciatic nerve, ultrasound

## Abstract

Deep gluteal syndrome (DGS) is a challenging cause of buttock pain and sciatic-like symptoms, often mimicking lumbar radiculopathy and remaining difficult to diagnose when conventional imaging is inconclusive. Structural abnormalities within the deep gluteal space, particularly at muscle-tendon insertions, may contribute to mechanically mediated sciatic nerve irritation but are frequently overlooked.

We report a case of DGS in a 45-year-old man, caused by piriformis enthesopathy with a cortically based bony spur at the greater trochanter, identified using computed tomography (CT) and functionally assessed with dynamic ultrasonography. CT allowed precise detection of the bony abnormality, while dynamic USG demonstrated reproducible motion-related changes at the piriformis enthesis during hip rotation, which may provide further information to a patient with DGS.

Targeted ultrasound-guided dextrose prolotherapy using a hyperosmolar dextrose solution was performed at the enthesopathic site (six sessions at two-week intervals), resulting in substantial and sustained symptom improvement at three-month follow-up. This case showed that the abnormal MRI findings may not be the cause of the patient's condition, and it highlights the diagnostic value of a multimodal imaging approach combining CT and dynamic ultrasound in this patient. Furthermore, it suggests that ultrasound-guided dextrose prolotherapy could be an effective, minimally invasive treatment option for selected patients with chronic, function-limiting DGS with symptomatic enthesopathy.

## Introduction

Deep gluteal syndrome (DGS) is a clinically challenging condition characterized by pain and dysesthesia in the buttock area, primarily caused by non-discogenic entrapment of the sciatic nerve within the deep gluteal space [1]. This syndrome predominantly affects individuals in their 40s and 50s, with a higher prevalence in females [2]. The clinical presentation of DGS often mimics lumbar radiculopathy or spinal stenosis, making differential diagnosis crucial [3]. While both conditions may cause lower limb weakness and paresthesia, a key distinguishing feature of DGS is the presence of pain-associated weakness during manual muscle testing, often indicative of an underlying tendinopathy or entrapment neuropathy [4].

The deep gluteal space is a complex anatomical compartment bounded superiorly by the inferior margin of the sciatic notch, inferiorly by the proximal origin of the hamstrings at the ischial tuberosity, medially by the sacrotuberous ligament and falciform fascia, laterally by the lateral lip of the linea aspera and the greater trochanter (GT), and roofed by the gluteus maximus [5]. It serves as a conduit for several neurovascular structures exiting through the greater and lesser sciatic foramina. While historical research has focused predominantly on sciatic nerve entrapment by the piriformis muscle, contemporary understanding recognizes a broader spectrum of etiologies, including various tendinopathies and enthesopathies within this space [4]. Our team has previously reported ligamentous calcification as a potential cause of DGS [6], and this case report contributes a new, specific structural etiology to this expanding paradigm: piriformis enthesopathy with bony spur formation

Diagnosing DGS remains a significant clinical challenge. Pathologies are often not visible on conventional imaging, such as standard radiographs, and even MRI frequently fails to demonstrate a definitive lesion corresponding to the patient’s symptoms [7]. Thus, DGS cannot be reliably confirmed or excluded based on MRI alone, underscoring the importance of a meticulous history and targeted physical examination. Given the diagnostic ambiguity and the potential role of mechanically mediated pain generators, a targeted regenerative injection strategy was considered. In this report, prolotherapy refers to a targeted regenerative injection strategy aimed at stabilizing pathological micro-motion at the enthesis and restoring altered local biomechanics. Hyperosmolar dextrose was used as the injectate in this prolotherapy protocol. We report a case of chronic DGS in a 45-year-old man, secondary to a bony spur at the piriformis insertion, identified on CT and functionally assessed with dynamic ultrasonography, and treated with ultrasound-guided dextrose prolotherapy, with substantial symptomatic improvement.

## Case presentation

A 45-year-old man presented to our clinic with a chief complaint of chronic left buttock pain of more than five years’ duration, accompanied by paresthesia in the left lower limb. The patient had no significant past medical history but had worked as a physical trainer and manual laborer. He reported no improvement despite previous interventions, including lumbar nerve blocks, physical therapy, and acupuncture for suspected lumbar disc disease. His pain severely limited his functional capacity, preventing him from working for more than 10 minutes at a time. Because of the severity and chronicity of his symptoms, he had been unable to maintain regular employment for almost five years and was receiving government welfare support at the time of presentation.

The pain was localized to the left hip and buttock, with radiating paresthesia down the posterior thigh. Symptoms were significantly aggravated by sitting or standing for more than ten minutes. Physical examination revealed a negative Straight Leg Raise Test (SLRT). However, several provocative tests for deep gluteal pain were positive, including the Seated Piriformis Test, Flexion, Adduction, and Internal Rotation (FAIR) test, Active Piriformis Test, and Modified Beatty Test. Motor strength testing of the lower extremities was within normal limits and without pain-induced weakness [4].

**Figure 1:**
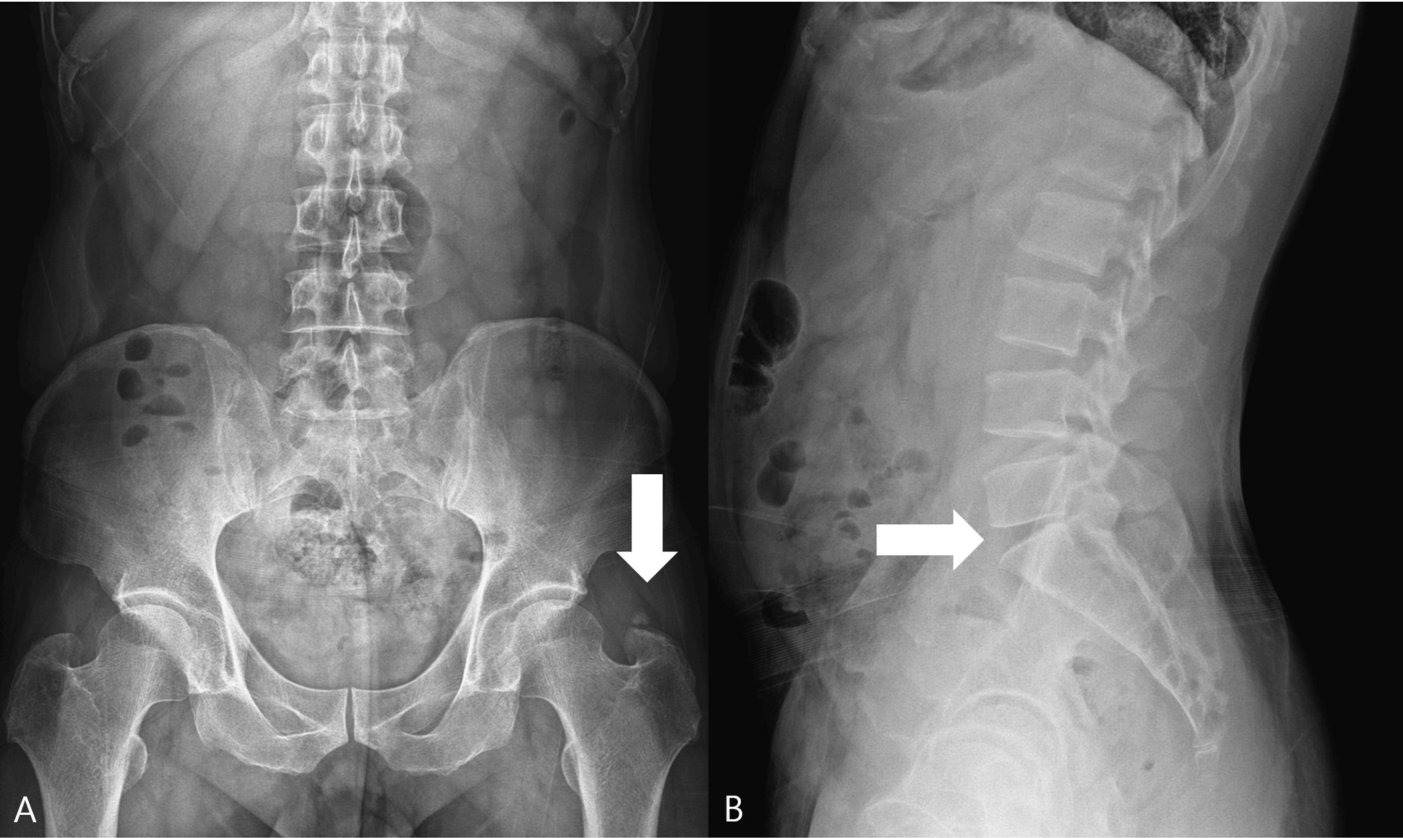
Lumbar spine radiographs. (A) Anteroposterior view showing calcification (arrow) near the left greater trochanter. (B) Lateral view demonstrating L5-S1 disc space narrowing (arrow).

An initial lumbar anteroposterior (AP) radiograph revealed calcification near the left greater trochanter (Figure 1A). A dedicated lumbar lateral radiograph demonstrated L5-S1 disc space narrowing (Figure 1B). On the AP radiograph, the opacity near the greater trochanter was interpreted as soft-tissue calcification rather than a discrete exostosis, and a bony spur could not be clearly identified.

**Figure 2:**
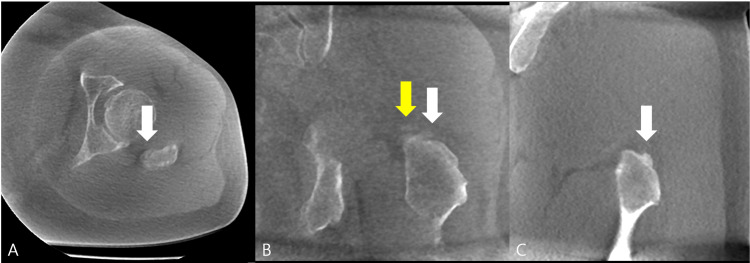
CT scan of the left hip. (A) Axial view demonstrating a bony spur (white arrow) arising from the posterior aspect of the greater trochanter. (B) Coronal view showing a separate calcified focus (yellow arrow) distinct from the bony spur (white arrow). (C) Sagittal view confirming that the bony spur (white arrow) is located on the posterosuperior facet of the greater trochanter.

**Figure 3:**
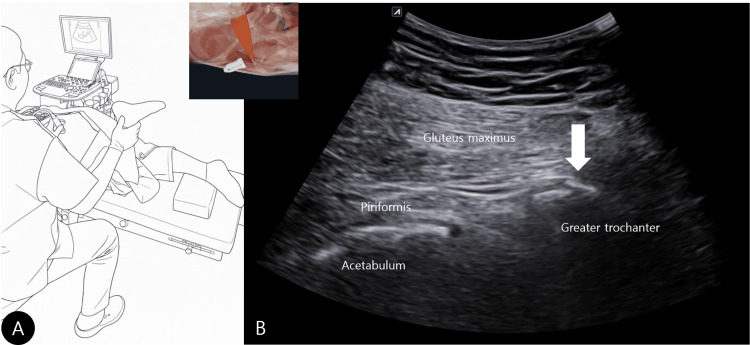
Dynamic ultrasonography of the left hip. (A) Schematic illustration of the prone position with the knee flexed to 90° for dynamic examination. (B) Before loading, the bony spur (white arrow) at the piriformis attachment is visualized; the ultrasound probe position is indicated in the upper left corner. Image credit: Yonghyun Yoon. Created by the authors based on an original clinical photograph (photograph not shown).

To further characterize this finding, a computed tomography (CT) scan of the hip was obtained. The axial (Figure 2A) and coronal view (Figure 2B), sagittal view (Figure 3C) clearly demonstrated a bony spur at the posterosuperior aspect of the greater trochanter, precisely at the insertion site of the piriformis muscle. Importantly, this CT-defined lesion was cortically based and continuous with the cancellous bone of the greater trochanter, clearly distinguishing it from the extra-osseous calcification seen on the lumbar radiograph. Focused musculoskeletal ultrasound further confirmed that the radiographic calcification and the CT-defined bony spur were anatomically separate structures. The presence of this bony spur on CT raised the suspicion of DGS secondary to piriformis enthesopathy.

Ultrasonography was performed with the patient prone to optimize exposure of the short external rotators; a pillow was placed under the abdomen, and another under the ankle to reduce hamstring-related tension. Diagnostic ultrasound visualized the bony spur at the greater trochanter. On static evaluation, the sciatic nerve demonstrated a symmetric cross-sectional area and normal echotexture compared with the contralateral side, indicating no overt nerve enlargement or compressive neuropathy. The attached muscle was then traced to confirm the piriformis, and the transducer was aligned along the long axis of the piriformis to localize the insertional site. Dynamic assessment was subsequently performed with the piriformis visualized along its long axis. With the proximal end of the transducer held stationary, internal and external rotation were applied with the knee flexed to 90° (Figure 3A) to load the insertion (Figure 3B), revealing reproducible relative motion at the piriformis enthesis during dynamic loading (Video 1).

**Video 1:** Dynamic examination of the left hip joint for the diagnosis of piriformis tendinopathy (long axis of the piriformis).

Despite the preservation of nerve diameter, additional dynamic examination of the short external rotators revealed that the sciatic nerve maintained its movement over the short external rotators, excluding the piriformis, during internal and external rotation, but the sciatic nerve on the affected side appeared to demonstrate less apparent excursion beneath the piriformis muscle compared with the contralateral side (Videos 2, 3) [8].

**Video 2:** Dynamic examination of the left hip joint for the diagnosis of piriformis tendinopathy (short axis of the piriformis).

**Video 3:** Dynamic examination of the left hip joint for the diagnosis of piriformis tendinopathy (contralateral side).

Given the imaging findings and clinical presentation, an ultrasound-guided dextrose prolotherapy procedure was planned with the dual intent of (1) providing diagnostic confirmation by eliciting concordant pain at the enthesopathic site and (2) delivering a therapeutic regenerative stimulus. While a diagnostic block with local anesthetic would represent an ideal confirmatory step, the prolotherapy injection was used to serve this combined diagnostic and therapeutic purpose within our clinical workflow.

**Figure 4:**
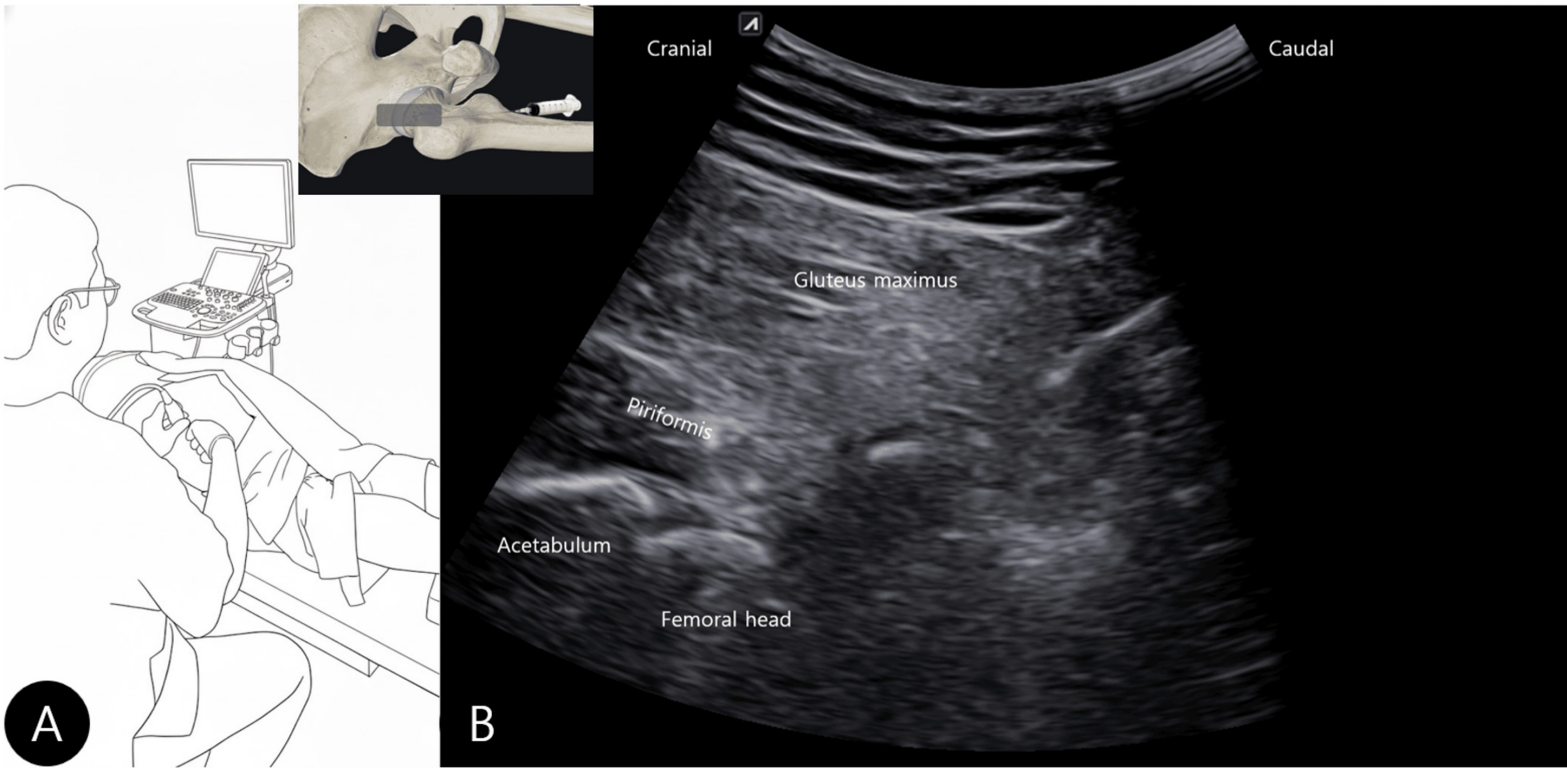
Ultrasound-guided dextrose prolotherapy injection at the piriformis enthesis. (A) Schematic illustration of the patient position and ultrasound transducer placement in an oblique sagittal plane aligned with the long axis of the piriformis muscle. (B) Real-time ultrasound image demonstrating needle advancement using a caudal-to-cranial in-plane approach toward the enthesopathic site, with the transducer maintained in the oblique sagittal orientation. The ultrasound probe position is indicated by a black rectangle in the upper center of (B), along with the injection direction. Image credit: Yonghyun Yoon. Created by the authors based on an original clinical photograph (photograph not shown).

The procedure was performed under real-time ultrasound guidance with the patient positioned as illustrated in the schematic drawing (Figure 4A). The ultrasound transducer was positioned in an oblique sagittal plane, aligned with the long axis of the piriformis muscle, to visualize the enthesopathic site and adjacent osseous landmarks. Using this probe orientation, a caudal-to-cranial in-plane approach was employed for needle advancement. A 10% hyperosmolar dextrose solution mixed with 0.2% lidocaine was injected precisely at the enthesopathic site, with real-time ultrasound confirming needle placement and target engagement (Figure 4B). The complete ultrasound-guided injection technique is demonstrated in Video 4. In Video 4, subtle micromotion of the targeted fragment is visible during needle contact and injection, and this finding is highlighted with a white arrow.

**Video 4:** Ultrasound-guided dextrose prolotherapy at the piriformis enthesis. The transducer is maintained in an oblique sagittal plane aligned with the long axis of the piriformis tendon, while the needle is advanced using a caudal-to-cranial in-plane approach to deliver 10% dextrose with 0.2% lidocaine at the enthesopathic site. Subtle micromotion of the targeted fragment during needle contact and injection is indicated by a white arrow. The moment of concordant pain reproduction when the needle tip touches the enthesopathic lesion is marked with a red arrow.

During injection, the patient’s characteristic buttock pain with radiating symptoms was reproduced, supporting concordance between the targeted enthesopathic lesion and the clinical pain generator. The patient subsequently underwent physical therapy three times weekly and performed daily seated isometric exercises targeting the deep gluteal musculature.

Because of financial constraints, more costly options (e.g., platelet-rich plasma injection or serial extracorporeal shockwave therapy) were not feasible. Prolotherapy sessions were repeated at two-week intervals.

To objectify treatment outcomes, standardized metrics were retrospectively estimated from contemporaneous clinical documentation because these instruments were not administered prospectively at fixed time points. Estimated visual analog scale (VAS) scores were derived from recorded patient-reported pain intensity descriptors, and estimated modified Harris Hip Score (mHHS) values were reconstructed from documented functional limitations and recovery milestones, including sitting tolerance and return-to-work status.

Pre-treatment, the patient's pain was consistently rated as severe and constant, correlating with an estimated VAS score of 8/10 [9]. His function was severely limited, corresponding to an estimated mHHS of 52, indicating poor hip function and major disability [10,11]. After six sessions of prolotherapy performed at two-week intervals over approximately 10 weeks, the patient reported more than 80% improvement in buttock pain and sciatic-like symptoms, with restoration of daily and work-related function. Three months after the final session, his symptoms correlated with an estimated VAS score of 2/10 and an estimated mHHS of 86, representing a transition to good function [12]. This magnitude of improvement (ΔVAS = -6, ΔmHHS = +34) aligns with meaningful clinical change reported in other interventional studies for chronic gluteal pain.

**Figure 5:**
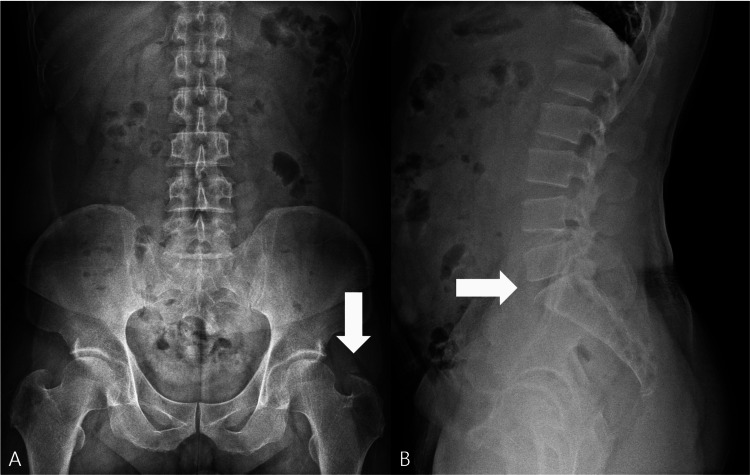
Follow-up lumbar spine X-ray at three months. (A) Anteroposterior view demonstrating persistent calcification (arrow) near the left greater trochanter, unchanged from prior studies. (B) Lateral view showing persistent disc space narrowing (arrow) at L5-S1, also unchanged.

Radiographs showed no change in the greater trochanteric calcification or L5-S1 disc space narrowing (Figures 5A-5B), consistent with symptomatic improvement attributable to functional stabilization and reduced irritation rather than morphologic resolution on imaging.

## Discussion

This case report presents several key clinical insights. First, it identifies enthesopathy of the piriformis at the greater trochanter as a primary, structurally identifiable cause of DGS. This finding underscores that the pathoanatomy of DGS extends beyond nerve entrapment to include intrinsic muscle-tendon pathologies, supporting a more nuanced model of the syndrome [4]. This aligns with the contemporary, expanded understanding of DGS, which emphasizes a spectrum of extra-spinal pathologies, including tendinopathy, enthesopathy, and adhesions as potential pain generators [4]. Second, it demonstrates the successful application of prolotherapy as a targeted treatment for this specific pathology. The use of a regenerative injection for this enthesopathy is conceptually supported by systematic review evidence for prolotherapy in managing sports-related tendinopathies [13]. Furthermore, it supports the diagnostic value of a multimodality imaging approach: CT effectively identified the bony spur, while dynamic ultrasonography demonstrated motion-related changes at the enthesis during dynamic loading, linking the structural anomaly to the functional pathology.

Our case adds a distinct etiological entity to the differential diagnosis of DGS. While our previous work highlighted sacrospinous ligament calcification as an extra-osseous, static cause of sciatic nerve irritation [6], the present case features a dynamic, intra-tendinous bony spur at a different anatomical site. This contrast illustrates that DGS can arise from diverse structural pathologies-both ligamentous and myotendinous-requiring tailored diagnostic and therapeutic approaches.

Anatomically, the sciatic nerve exits the greater sciatic foramen and passes through the deep gluteal space in close proximity to the piriformis tendon and its insertion on the greater trochanter. The enthesopathy may cause abnormal muscle tone or shear force of piriformis and the perineurial tissues structure during dynamic hip motion, particularly in flexion and internal rotation, provoking buttock and posterior thigh pain in our case. In addition, dynamic ultrasonographic examination of the piriformis can visualize the spur, the enthesis, and the sciatic nerve excursion beneath the piriformis much better than static CT and MRI. Taken together, these findings suggest a possible mechanism of mechanically mediated irritation arising from enthesopathy rather than chronic compressive neuropathy.

The diagnosis of DGS is often one of exclusion. While MRI is the preferred advanced imaging modality for soft tissue assessment, its sensitivity for extra-pelvic sciatic nerve entrapment is limited [7]. Conversely, although CT imaging is inherently limited in evaluating the sciatic nerve and therefore cannot independently diagnose DGS, it is superior for detecting bony abnormalities [14]. In our case, CT served as a valuable adjunct by clearly identifying the enthesopathic spur, providing structural information that conventional MRI could not offer. The precise localization of the spur at the piriformis insertion on CT established a strong anatomic correlation with the patient’s symptoms. Subsequently, dynamic ultrasound examination confirmed functionally relevant motion-related findings at the enthesis, an assessment beyond the capabilities of static CT or MRI [15]. This multimodal imaging strategy strengthened diagnostic confidence and enabled accurate targeting for intervention.

The management of DGS typically involves a stepwise approach, beginning with conservative measures such as physical therapy, activity modification, and analgesic medications [16]. When these fail, interventional procedures are considered. Common injections include corticosteroids or local anesthetics [17]. However, dextrose prolotherapy offers a regenerative alternative. Through prolotherapy using a hyperosmolar dextrose solution, a localized regenerative response may be stimulated, and a localized inflammatory healing response may strengthen ligaments and tendons, potentially decompressing adjacent neural structures by stabilizing the irritant focus [6,13,18,19]. The proposed mechanism extends beyond mechanical stabilization. Enthesopathies are characterized by pathological neurovascular invasion at the bone-tendon junction, which is a significant source of pain. Prolotherapy, by promoting organized tissue repair and reducing degenerative changes, may help modulate this aberrant innervation and neovascularization, thereby addressing a fundamental pain generator in chronic enthesopathic conditions [20,21].

Dextrose prolotherapy was chosen in this case as a pragmatic regenerative option directly targeting the piriformis enthesis and the unstable spur. Although prolotherapy has traditionally been described for myotendinous and ligamentous disorders, it is conceptually well suited for enthesopathic lesions, where pathological micro-motion at the bone-tendon junction can serve as a persistent nociceptive and mechanical irritant. By promoting localized tissue remodeling and stabilizing the enthesis, prolotherapy may reduce abnormal motion of the spur and dampen inflammatory signaling around the sciatic nerve. The clinical outcome in this case, an estimated improvement from a VAS of 8 to 2 and an mHHS from 52 to 86, represents a substantial therapeutic effect. This magnitude of improvement is comparable to outcomes reported in other interventional case series for DGS, such as a mean VAS reduction from 7.2 to 1.6 following endoscopic sciatic nerve decompression [3].

In addition, the patient’s welfare status and inability to afford platelet-rich plasma (PRP) or serial extracorporeal shockwave therapy (ESWT) made dextrose prolotherapy an ethically appropriate and financially feasible choice.

Moreover, the use of ultrasound guidance was pivotal. It allowed for not only real-time, accurate needle placement into the enthesopathic lesion but also capitalized on the prior dynamic examination that had confirmed the site's pathology. During the intervention, needle guidance was optimized by maintaining the transducer in an oblique sagittal plane aligned with the course of the piriformis tendon. This probe orientation facilitated simultaneous visualization of the enthesopathic site and adjacent osseous landmarks, enabling precise targeting using a caudal-to-cranial in-plane needle trajectory.

However, this is, to our knowledge, the first reported case specifically targeting a piriformis muscle enthesopathy with prolotherapy, leading to a successful outcome in a patient with a five-year history of debilitating pain. The reproduction of the patient's pain during the injection and the significant clinical improvement afterward strongly support the causal role of this enthesopathy.

Limitations

This report has several limitations inherent to its design. First, the diagnosis relied on imaging correlation and pain reproduction during injection rather than a confirmatory diagnostic block with local anesthetic, which is considered a more definitive method for identifying a pain generator [22]. Second, outcome assessment was initially based on subjective patient reporting. The use of validated, patient-reported outcome measures (e.g., VAS for pain and Hip Disability and Osteoarthritis Outcome Score) at predefined intervals would have provided more robust and generalizable data on treatment efficacy [23]. The VAS and mHHS scores were estimated retrospectively to provide standardized metrics; prospective collection would be more robust. Finally, as a single case report, it demonstrates proof of concept but cannot establish treatment efficacy or generalizability. Larger, controlled studies are needed to validate this approach.

## Conclusions

This case highlights that in patients with clinical signs of DGS but inconclusive MRI findings, a multimodality imaging approach can be critical. CT is excellent for identifying bony enthesopathies, while dynamic ultrasonography can confirm instability and provide functional correlation. A bony spur at the insertion of the piriformis should be considered a potential source of sciatic nerve irritation. Ultrasound-guided prolotherapy, leveraging both real-time targeting and insights from the dynamic exam, directed at this specific lesion represents an effective and minimally invasive treatment option, capable of providing substantial and rapid relief even in long-standing, work-limiting cases.
